# An in vitro assay system for antihyperlipidemic agents by evaluating lipoprotein profiles from human intestinal epithelium-like cells

**DOI:** 10.1007/s13205-012-0085-1

**Published:** 2012-09-05

**Authors:** Junichiro Takahashi, Kikumi Ogihara, Yuko Naya, Fumiko Kimura, Mizuho Itoh, Yuka Iwama, Yukie Matsumoto, Gen Toshima, Keishi Hata

**Affiliations:** 1 Skylight Biotech Inc., 100-4 Sunada, Iijima, Akita 010-0911 Japan; 2 School of Life and Environmental Science, Azabu University, 1-17-71 Fuchinobe, Chuo-ku, Sagamihara, Kanagawa 252-5201 Japan; 3 Akita Research Institute of Food and Brewing, 4-26 Sanuki, Araya-machi, Akita 010-1623 Japan

**Keywords:** Caco-2 cell differentiation, Intestinal cells, Lipoprotein profile, Triglyceride, Antihyperlipidemic agent

## Abstract

We developed an in vitro screening system for antihyperlipidemic activity by measuring lipoprotein profiles secreted from human intestinal epithelium-like cells from the colon cancer cell line, Caco-2. Sodium (Na) butyrate at 5 mM differentiated Caco-2 cells into intestinal epithelium-like cells and numerous microvilli on the apical side of cells were observed under transmission electron microscopy. Real-time RT-PCR analysis revealed that Na butyrate stimulated expression levels of intestinal differentiation markers in Caco-2 cells in a dose-dependent manner and 5 mM Na butyrate up-regulated intestinal alkaline phosphatase, sucrase–isomaltase complex, and microsomal triglyceride transfer protein by 8.1-, 1.9-, and 2.1-fold that of non-treated cells, respectively. Lipoprotein secretions from differentiated Caco-2 cells were promoted by lysophosphatidyl choline and Na oleate, which are a stimulator of lipoprotein secretion and a substrate of triglycerides, respectively. We examined the effects of Pluronic L-81, a lipoprotein secretion inhibitor, on lipoprotein profiles of differentiated Caco-2 cells. Pluronic L-81 at 1.0 μg/ml inhibited TG contents in lipoprotein fractions from cells by 25.6 % and secretion was completely suppressed by the agent at 10 μg/ml.

**Electronic supplementary material:**

The online version of this article (doi:10.1007/s13205-012-0085-1) contains supplementary material, which is available to authorized users.

## Introduction

Excessive intakes of dietary sugars and lipids such as triglycerides (TG) and cholesterol cause visceral fat accumulation leading to metabolic syndrome, which includes glucose intolerance, hypertension, dyslipidemia, and obesity. After dietary TG is cleaved into fatty acids and 2-mono-glycerides by gastric and pancreatic lipase, free fatty acids are incorporated into micelles with cholesterol, bile acids, and phosphatidylcholine (PC), which are then absorbed into intestinal epithelium cells. In intestinal epithelium cells, TG synthesized from fatty acids and cholesterol are packed into lipoproteins and secreted into lymphatic vessels. The inhibition of lipid transport in intestinal epithelium cells is effective for improving metabolic syndrome. Ezetimibe, which is a cholesterol transporter inhibitor, was administered to hypercholesterolemia patients (Clader 2005; Salen et al. 2004). In many cases, experimental animals such as the hyperlipidemic mouse/rat have been used for screening anti-metabolic syndrome activities; however, studies using experimental animals are very expensive and it is difficult to evaluate many test samples at one time. We previously developed a novel screening system for antihyperlipidemic agents by assessing the profile of lipoproteins secreted from hepatoma cells after separation by HPLC and evaluated antihyperlipidemic agents or activities in foodstuffs (Itoh et al. 2009; Takahashi et al. 2011).

The human colon cancer cell line, Caco-2, is known to differentiate spontaneously or chemically into intestinal epithelium-like cells and any nutrient transporters such as minerals (Han et al. 1999), glucose (Harris et al. 1992), and amino acids (Vermeulen et al. 2011) were expressed through cell differentiation. Therefore, differentiated Caco-2 cells have been frequently used as an in vitro transport model of nutrients (Faria et al. 2009). It was reported that lipid absorption and lipoprotein secretions in the basolateral side of differentiated Caco-2 cells were observed using ^3^H-labeled oleic acid and ^14^C-labeled cholesterol (Nakano et al. 2009). In the present study, we evaluated lipoprotein secretions from chemically differentiated Caco-2 cells under optimum concentrations of sodium (Na) oleate and lysophosphatidyl choline (lysoPC) without the use of radioactive compounds.

## Materials and methods

### Chemicals and cell line

Cholesterol-free bovine serum albumin (BSA), lysoPC from egg yolks, and Na oleate were purchased from Wako Pure Chemical Industry. Pluronic L-81 was from BASF Co. Ltd., and the human colon cancer cell line, Caco-2, was obtained from RIKEN Cell Bank.

### Differentiation of Caco-2 cells on ThinCert

Caco-2 cells were seeded at a density of 2.5 × 10^5^ cells per well in a 12-well ThinCert (12-mm diameter and 8.0-μm pore size, Greiner) precoated with collagen and cultured for 2 days in Dulbecco’s modified Eagle’s medium (DMEM) containing 10 % fetal bovine serum (FBS) and 100 U/ml penicillin—100 mg/ml streptomycin. In Caco-2 cell preculture on ThinCert for 2 days, cells reached to confluent monolayer. The differentiation of Caco-2 cells on ThinCert membranes was induced by treatments of 0–5 mM Na butyrate for 4 days.

### Transmission electron microscopy (TEM)

Caco-2 cells in ThinCert were fixed with 0.25 % glutaraldehyde/phosphate buffer (PB), rinsed with PB, and post-fixed with 2.0 % osmium tetroxide. Cells were dehydrated by ethyl alcohol according to a routine technique, displaced with QY-1, and embedded in Epon. Ultrathin sections of cells were carried out and cells were double stained with uranyl acetate and lead citrate and observed under a JEM-1400 electron microscope.

### Lipoprotein secretion from differentiated Caco-2 cells

**Fig. 1:**
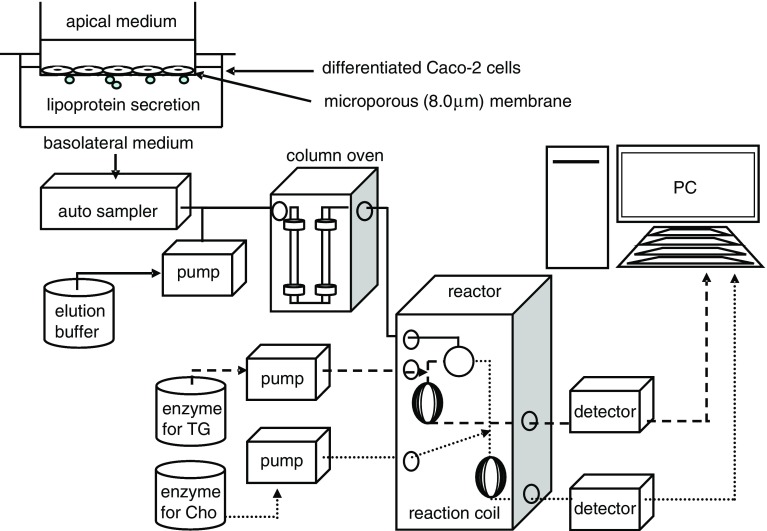
HPLC system with online enzymatic dual detection of TG and cholesterol of lipoproteins in the basolateral medium of differentiated Caco-2 cells

LysoPC and/or Na oleate were added in the apical medium (DMEM containing 1.0 % BSA, 1 ml) of differentiated Caco-2 cells, and secreted lipoproteins in the basolateral medium (DMEM containing 1.0 % BSA, 500 μl) were measured by LipoSEARCH^®^ (Fig. 1).

### Lipoprotein profile (LipoSEARCH^®^)

The separation and determination of four major classes of lipoprotein (chylomicron; CM, very low density lipoprotein; VLDL, LDL, and high density lipoprotein; HDL) were performed as previously described (Itoh et al. 2009). Briefly, lipoproteins from 80 μl of culture media were separated using a gel permeation HPLC system (LipoSEARCH^®^). Effluents were continuously monitored at 550 nm after an online enzymatic reaction using diacolor liquid TG-S (TOYOBO Co. Ltd.) for triglycerides and a Cholescolor liquid kit for cholesterol (TOYOBO Co. Ltd.). Triglyceride and cholesterol concentrations in the four major lipoproteins were calculated using frozen serum-based standard material (Kyowa Kirin Co. Ltd.) as a standard and our in-house computer program.

### RNA extraction and cDNA synthesis

Total RNA was isolated form Caco-2 cells using QuickGene RNA culture cell kit S (FUJIFILM, Co.). Template cDNA synthesis was performed with 5 μg of total RNA using the PrimeScript RT reagent kit (TAKARA Bio, Inc.)

### Real-time RT-PCR

In a fluorescent temperature cycler (Chromo4; Bio-Rad Laboratories, Inc.), 2.5 % of each RT reaction solution was amplified in 25 μl of 1× SYBR Premix Ex Taq II (TAKARA Bio, Inc.) containing 0.2 μM of each primer. Samples were incubated in the thermal cycler for an initial denaturation at 95 °C for 10 s, followed by 40 PCR cycles. Each cycle consisted of 95 °C for 5 s and 60 °C for 30 s. The oligonucleotide primers used in the experiment are indicated in Table 1. To confirm the amplification of specific transcripts, melting curve profiles (cooling the sample to 60 °C and heating slowly to 95 °C with the continuous measurement of fluorescence) were produced at the end of each PCR. The relative expression level of both mRNAs was normalized by the amount of glyceraldehyde-3-phosphate dehydrogenase (GAPDH) mRNA (forward primer, 5′-GCACCGTCAAGGCTGAGAAC-3′; reverse primer, 5′-TGGTGAAGACGCCAGTGGA-3′).

**Table 1:** Effects of sodium butyrate on mRNA expressions in Caco-2 cells

Gene name	Sequences for oligonucleotide primer		Relative intensity/GAPDH (-fold)			
			Na butyrate (mM)			
	Forward	Reverse	0	1	2	5
IAP	5′-GCAACCCTGCAACCCACCCAAGGAG -3′	5′-CCAGCATCCAGATGTCCCGGGAG-3′	1.0 ± 0.1	2.3 ± 0.1**	3.3 ± 0.3**	8.2 ± 0.1**
SI	5′-GACAACTATGCACGATGGGACAAC-3′	5′-TTGCATCCAGCGGGTACAGA-3′	1.0 ± 0.1	1.1 ± 0.1	1.1	1.5 ± 0.1**
ApoB	5′-TCGCCTGCCAAACTGCTTC-3′	5-CATTGGTGCCTGTGTTCCATTC-3′	1.0 ± 0.1	1.0 ± 0.1	1.1 ± 0.3	1.1 ± 0.1
MTTP	5′-TCTCTACTCGGGTTCTGGCATTCTA-3′	5′-GCTGCGATTAAGGCTTCCAGTC-3′	1.0 ± 0.2	1.4 ± 0.2	1.9 ± 0.3*	1.8 ± 0.1*
Cldn-1	5′-GCATGAAGTGTATGAAGTGCTTGGA-3′	5′-CGATTCTATTGCCATACCATGCTG-3′	1.0	1.3 ± 0.1*	1.4 ± 0.2*	1.8 ± 0.2**
Occludin	5′-TCCTATAAATCCACGCCGGTTC-3′	5′-CTCAAAGTTACCACCGCCTGCTG-3′	1.0 ± 0.1	1.2 ± 0.2	1.5 ± 0.1**	2.1 ± 0.1**
PCNA	5′-GGCCGAAGATAACGCGGATAC-3′	5′-GGCATATACGTGCAAATTCACCAG-3′	1.0	0.8**	0.5**	0.5 ± 0.1**

Caco-2 cells on ThinCert membranes were treated with or without various concentrations of Na butyrate for 4 days and mRNA expression was analyzed by real-time RT-PCR

Data are expressed relative to untreated control cells and represent mean ± SD (*n* = 4, * *p* < 0.05, ** *p* < 0.01 vs. untreated cells)

### Statistical analysis

Data are expressed as the mean ± standard deviation (SD). The significance of differences was analyzed using a one-way ANOVA with the Dunnett’s (Tables 1, 2, S1 and S2, and Fig. 3) and Tukey’s (Fig. 4) multiple comparison test. A value of *p* < 0.05 was considered to be significant.

**Table 2:** Effect of Pluronic L-81 on TG and cholesterol secretion from differentiated Caco-2 cells

	Pluronic-L81 (μg/ml)			
	0	0.1	1.0	10
Cell number (×10^5^)	2.8 ± 0.2	2.7	2.7 ± 0.2	2.5 ± 0.2
Triglycerides (μg/10^6^ cells)				
Total	46.1 ± 10.1	42.2 ± 5.9	11.1 ± 1.8**	0**
CM	0.6 ± 0.2	0.7 ± 0.1	0**	0**
VLDL	37.1 ± 9.1	33.8 ± 4.9	5.6 ± 1.2**	0**
LDL	7.8 ± 0.9	7.2 ± 1.9	5.1 ± 0.7**	0**
HDL	0.5 ± 0.1	0.5 ± 0.2	0.4	0**
Cholesterol (μg/10^6^ cells)				
Total	3.8 ± 0.5	3.5 ± 0.4	1.4 ± 0.1**	1.3 ± 0.1**
CM	0.4	0.2	0.3 ± 0.1	0.5 ± 0.4
VLDL	2.5 ± 0.4	2.7 ± 0.3	0.7 ± 0.1**	0.5 ± 0.4**
LDL	0.7 ± 0.1	0.6 ± 0.1	0.4 ± 0.1*	0.1 ± 0.1**
HDL	0.2	0	0.2	0.2

Differentiated Caco-2 cells were cultured in serum-free DMEM containing 1.0 % BSA with or without 1–10 μg/ml Pluronic L-81 for 4 days and the levels of triglycerides and cholesterol in the basolateral medium were determined

Data represent mean ± SD (*n* = 4)

* *p* < 0.05; ** *p* < 0.01 versus untreated control cells

## Results and discussion

### Differentiation of Caco-2 cells into intestinal epithelium-like cells

**Fig. 2:**
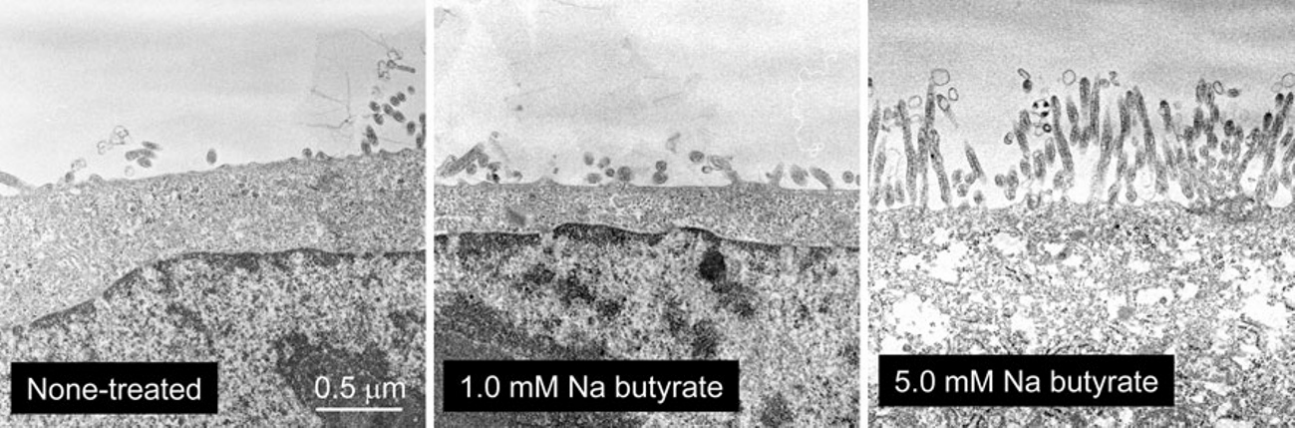
Caco-2 cell differentiation into intestinal epithelium-like cells by Na butyrate. Following treatment of Caco-2 cells without or with 1.0 and 5.0 mM Na butyrate for 4 days, microvilli on the apical side of cells were observed under TEM

Briefly, we confirmed whether some concentrations of Na butyrate morphologically and functionally differentiate Caco-2 cells into intestinal epithelium-like cells in vitro. TEM observations showed that Na butyrate treatments at 5 mM promoted microvilli formation in the apical side of Caco-2 cells relative to non-treated or 1 mM treated cells (Fig. 2). Real-time RT-PCR analysis (Table 1) revealed that intestinal alkaline phosphatase (IAP) and sucrase–isomaltase complex (SI), which are hallmarks of intestinal cell differentiation, were up-regulated by Na butyrate in a dose-dependent manner. Microsome triglyceride transfer protein (MTTP), which is localized to the lumen of the endoplasmic reticulum in the liver and intestine (Hussain et al. 2003) and is necessary for the assembly of apolipoprotein B (ApoB), was elevated by Na butyrate at over 2 mM; however, Na butyrate treatment did not affect the expression of ApoB mRNA. Tight junctions play an important role in the barrier function of the intestine and other tissues and some tight junctional proteins were induced and distributed in differentiated Caco-2 cell borders (Schlegel et al. 2010). The expressions of two tight junctional proteins, claudin (Cldn)-1 and occludin, were elevated up to approximately twofold by 5 mM Na butyrate. Furthermore, Na butyrate treatment attenuated the expression of proliferating cell nuclear antigen (PCNA), a cell-cycle regulator expressed in the nucleus of proliferating cells. In some tumors, the degree of PCNA expression has been correlated with tumor progression and grade (Zeng and Davis 2003). These results suggested that Na butyrate morphologically and functionally stimulated Caco-2 cell differentiation into intestinal epithelium-like cells in vitro. In particular, 5 mM Na butyrate treatment induced numerous microvilli formation and specific proteins for intestinal epithelium cells at the mRNA level. We used Caco-2 cells treated with 5 mM Na butyrate for 4 days as an intestinal epithelium cell model in the following experiments.

### Lipoproteins released from differentiated Caco-2 cells

**Fig. 3:**
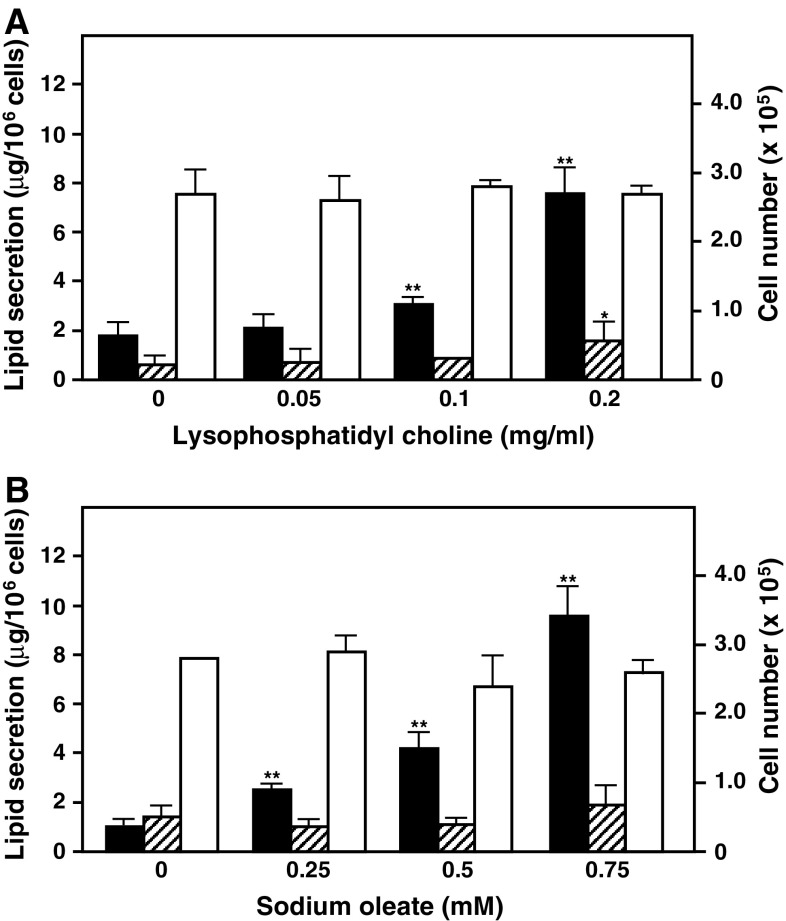
Effects of lysoPC and Na oleate on lipoprotein secretions from differentiated Caco-2 cells. Differentiated Caco-2 cells were cultured in serum-free DMEM containing 1.0 % BSA, 0.75 mM Na oleate, and some concentrations of lysoPC for 2 days (**a**), and in serum-free DMEM containing 1.0 % BSA, 0.2 mg/ml lysoPC, and some concentrations of Na butyrate for 2 days (**b**), and the levels of total TG (*black bar*) and cholesterol (*hatched bar*) in the basolateral medium and viable cell numbers (*white bar*) were determined. Data represent mean ± SD (*n* = 4). *p < 0.05; **p < 0.01 versus control cells

**Fig. 4:**
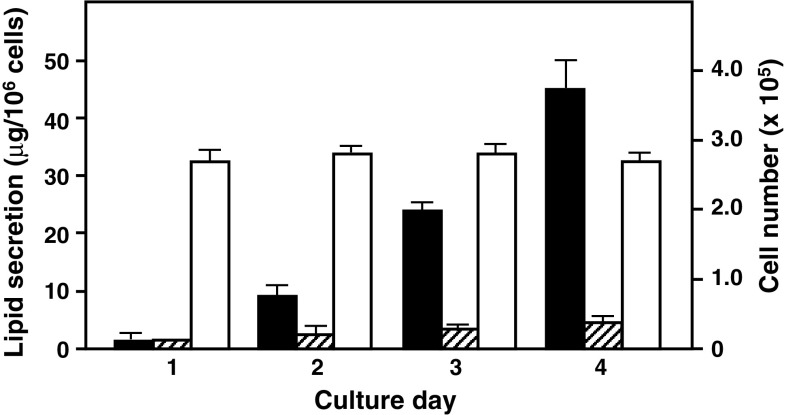
Time course of TG and cholesterol secretion from differentiated Caco-2 cells. Differentiated Caco-2 cells were cultured in serum-free DMEM containing 1.0 % BSA, 0.2 mg/ml lysoPC, and 0.75 mM Na oleate for the appointed period and the levels of total TG (*black bar*) and cholesterol (*hatched bar*) in the basolateral medium and viable cell numbers (*white bar*) were determined. Data represent mean ± SD (*n* = 4)

LysoPC was found to enhance lipoprotein secretion from differentiated Caco-2 cells by accelerating lipid absorption, assembly, and clearance (Nakano et al. 2009) and Na oleate, a component of TG, stimulated TG synthesis and secretion in the intestinal epithelium or hepatocyte/hepatoma cells (Traber et al. 1987; Homan et al. 1991). We studied the optimum concentrations of lysoPC and Na oleate on lipoprotein secretion from differentiated Caco-2 cells. LysoPC at over 0.1 mg/ml stimulated TG secretion from cells in the presence of 0.75 mM Na oleate and 0.2 mg/ml lysoPC elevated TG and cholesterol secretion by 4.2- and 2.7-fold, respectively, over that from differentiated Caco-2 cells treated with Na oleate alone (Fig. 3a and Table S1). Na oleate promoted TG secretion in a dose-dependent manner from differentiated Caco-2 cells under 0.2 mg/ml lysoPC (Fig. 3b and Table S2); however, more than 0.5 mg/ml lysoPC and more than 1.0 mM Na butyrate exhibited cytotoxicity toward differentiated Caco-2 cells and lipoprotein secretion from cells could not be determined at those doses. Furthermore, we investigated time-dependent lipid secretions in the basolateral medium of differentiated Caco-2 cells. Lipid secretions from differentiated Caco-2 cells in the presence of Na oleate and lysoPC were significantly accelerated in the incubation period for 4 days (Fig. 4). In particular, TG and cholesterol in VLDL and LDL fractions mainly increased in the incubation period; however, their levels in CM and HDL were very low (Table S3).

**Fig. 5:**
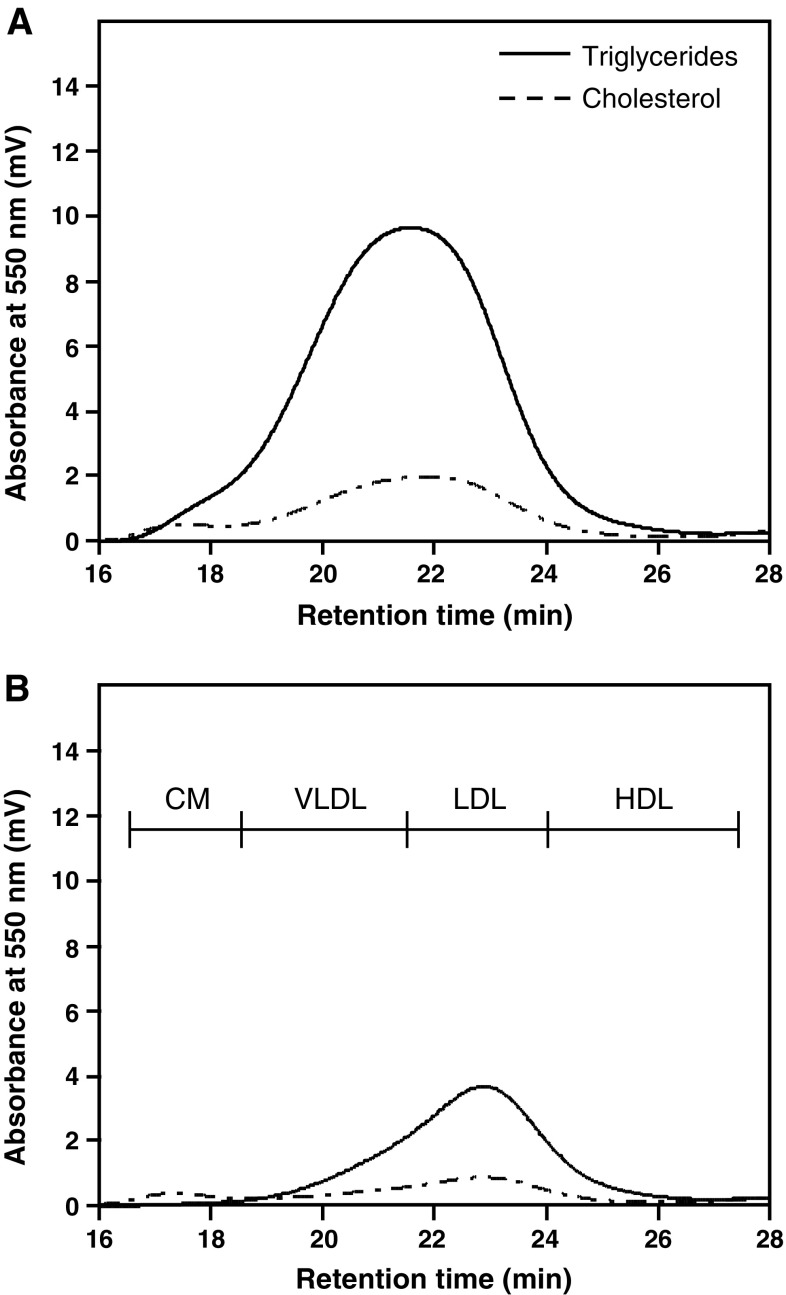
Lipoprotein profile from differentiated Caco-2 cells treated with Pluronic L-81. Differentiated Caco-2 cells were cultured in serum-free DMEM containing 1.0 % BSA, 0.2 mg/ml lysoPC, and 0.75 mM Na oleate without (**a**) or with 1.0 μg/ml Pluronic L-81 (**b**) for 4 days and lipoprotein profiles from cells were measured

Pluronic L-81 has been found to inhibit lipoprotein secretion from differentiated Caco-2 cells (Fatma et al. 2006) and we studied the effect of Pluronic L-81 at 0.1–10 μg/ml on lipid secretion (Table 2). Figure 5 shows the effects of the agent at 1.0 μg/ml on lipoprotein profiles from differentiated Caco-2 under Na oleate and lysoPC. Pluronic L-81 at 1.0 μg/ml reduced TG and cholesterol secretion from differentiated Caco-2 cells by 24.0 and 36.8 %, respectively, relative to untreated cells.

In the present study, we developed an evaluation system for lipoprotein secretion from intestinal epithelium-like cells without the use of radioactive compounds. This assay system is useful for the study of lipid absorption in the intestine and screening for antihyperlipidemia agents to target intestinal lipid transport.

## Electronic supplementary material

Below is the link to the electronic supplementary material.

Supplementary material 1 (DOC 121 kb)
