# Supplementary material for: An in vitro assay system for antihyperlipidemic agents by evaluating lipoprotein profiles from human intestinal epithelium-like cells
Source: 3 Biotech. 2012 Sep 5;3(3):213–8. doi: 10.1007/s13205-012-0085-1 (PMC3646105; doi:10.1007/s13205-012-0085-1)
Supplement: Supplementary file 1 — Supplementary material 1 (DOC 121 kb) [file 13205_2012_85_MOESM1_ESM.doc]

An in vitro assay system for antihyperlipidemic agents by evaluating lipoprotein profiles from human intestinal epithelium-like cells

Junichiro Takahashi et al.

Supplementary materials (Tables)

Contents

Table S1.

Effect of lysoPC on TG and cholesterol secretion from differentiated Caco-2 cells.

Table S2.

Effect of Na oleate on TG and cholesterol secretion from differentiated Caco-2 cells

Table S3.

Time course of TG and cholesterol secretion from differentiated Caco-2 cells

**Table S1**

**Effect of lysoPC on TG and cholesterol secretion from differentiated Caco-2 cells**

|  | LysoPC (mg/ml) | | | |
| --- | --- | --- | --- | --- |
|  | 0 | 0.05 | 0.1 | 0.2 |
| Cell number (x 10^5^) | 2.7±0.4 | 2.6±0.4 | 2.8±0.2 | 2.7±0.1 |
|  |  |  |  |  |
| Triglycerides (μg/10^6^ cells) |  |  |  |  |
| Total | 1.8±0.4 | 2.1±0.7 | 3.1±0.4** | 7.6±1.3** |
| CM | 0 | 0 | 0 | 0.2±0.2 |
| VLDL | 0 | 0.5±0.8 | 1.0±0.3** | 4.6±1.0** |
| LDL | 0 | 0.2±0.4 | 0.5±0.1** | 1.5±0.2* |
| HDL | 1.8±0.4 | 1.6±0.3 | 1.5±0.2 | 1.3±0.4 |
|  |  |  |  |  |
| Cholesterol (μg/10^6^ cells) |  |  |  |  |
| Total | 0.6±0.3 | 0.7±0.4 | 0.9 | 1.6±0.9* |
| CM | 0 | 0 | 0 | 0.2±0.1 |
| VLDL | 0.3±0.3 | 0.4±0.4 | 0.4±0.1 | 0.6±0.4 |
| LDL | 0.3 | 0.2± | 0.3 | 0.4±0.4 |
| HDL | 0.1 | 0.1±0.2 | 0.2 | 0.4** |

Differentiated Caco-2 cells were cultured in serum-free DMEM containing 1.0% BSA, some concentrations of lysoPC, and 0.75 mM Na oleate for 2 days and the levels of triglycerides and cholesterol in the basolateral medium were determined. Data represent means ± SD (n = 4). * *p* < 0.05. ** *p* < 0.01 versus control cells.

**Table S2**

**Effect of Na oleate on TG and cholesterol secretion from differentiated Caco-2 cells**

|  | Na oleate (mM) | | | |
| --- | --- | --- | --- | --- |
|  | 0 | 0.25 | 0.5 | 0.75 |
| Cell number (x 10^5^) | 2.8 | 2.9±0.2 | 2.4±0.5 | 2.6±0.1 |
|  |  |  |  |  |
| Triglycerides (μg/10^6^ cells) |  |  |  |  |
| Total | 1.0±0.1 | 2.5±0.1** | 4.2±0.4** | 9.6±0.9** |
| CM | 0 | 0 | 0 | 0.3±0.1 |
| VLDL | 0.1±0.1 | 1.0±0.1** | 2.2±0.3** | 5.8±0.6** |
| LDL | 0.2 | 0.8±0.1* | 1.3±0.2** | 3.0±0.4** |
| HDL | 0.7 | 0.7 | 0.7±0.1 | 0.5±0.1 |
|  |  |  |  |  |
| Cholesterol (μg/10^6^ cells) |  |  |  |  |
| Total | 1.4±0.4 | 1.0±0.5 | 1.1±0.1 | 1.9±0.3 |
| CM | 0.2±0.4 | 0 | 0 | 0.2±0.1 |
| VLDL | 0.4±0.1 | 0.6±0.6 | 0.4±0.1 | 0.8±0.1 |
| LDL | 0.4±0.1 | 0.2 | 0.5±0.1 | 0.7±0.2 |
| HDL | 0.4±0.3 | 0.2±0.2 | 0.2 | 0.2±0.1 |

Differentiated Caco-2 cells were cultured in serum-free DMEM containing 1.0% BSA, 0.2 mg/ml lysoPC, and some concentrations of Na oleate for 2 days and the levels of triglycerides and cholesterol in the basolateral medium were determined. Data represent means ± SD (n = 4). * *p* < 0.05. ** *p* < 0.01 versus control cells.

**Table S3**

**Time course of TG and cholesterol secretion from differentiated Caco-2 cells**

|  | Culture day | | | |
| --- | --- | --- | --- | --- |
|  | 1 | 2 | 3 | 4 |
| Cell number (x 10^5^) | 2.7±0.2 | 2.8±0.2 | 2.8±0.3 | 2.7±0.1 |
|  |  |  |  |  |
| Triglycerides (μg/10^6^ cells) |  |  |  |  |
| Total | 1.4±0.8 | 9.0±1.4 | 23.9±1.3 | 44.9±5.7 |
| CM | 0 | 0 | 0 | 0.2 |
| VLDL | 0.8±0.4 | 5.7±0.8 | 16.2±1.4 | 33.0±4.0 |
| LDL | 0.6±0.2 | 3.2±0.6 | 7.3±0.2 | 10.8±1.5 |
| HDL | 0 | 0.1±0.2 | 0.4 | 0.9±0.2 |
|  |  |  |  |  |
| Cholesterol (μg/10^6^ cells) |  |  |  |  |
| Total | 1.5 | 2.4±0.2 | 3.5±0.2 | 4.5±0.2 |
| CM | 0.4 | 0.4 | 0.4 | 0.4 |
| VLDL | 0.5±0.2 | 1.0±0.2 | 1.9 | 2.3±0.4 |
| LDL | 0.4 | 0.8 | 1.1 | 1.4±0.2 |
| HDL | 0.1±0.2 | 0.3±0.2 | 0.4 | 0.4 |

Differentiated Caco-2 cells were cultured in serum-free DMEM containing 1.0% BSA, 0.2 mg/ml lysoPC, and 0.75 mM Na oleate for the appointed period and the levels of triglycerides and cholesterol in the basolateral medium were determined. Data represent means ± SD (n = 4).
